# Supplementary material for: Electric shock causes a fleeing-like persistent behavioral response in the nematode Caenorhabditis elegans
Source: Genetics. 2023 Aug 18;225(2):iyad148. doi: 10.1093/genetics/iyad148 (PMC10550322; doi:10.1093/genetics/iyad148)
Supplement: iyad148_Supplementary_Data [file iyad148_supplementary_data.zip › Figure_S1_GENETICS-2022-305494.pdf]

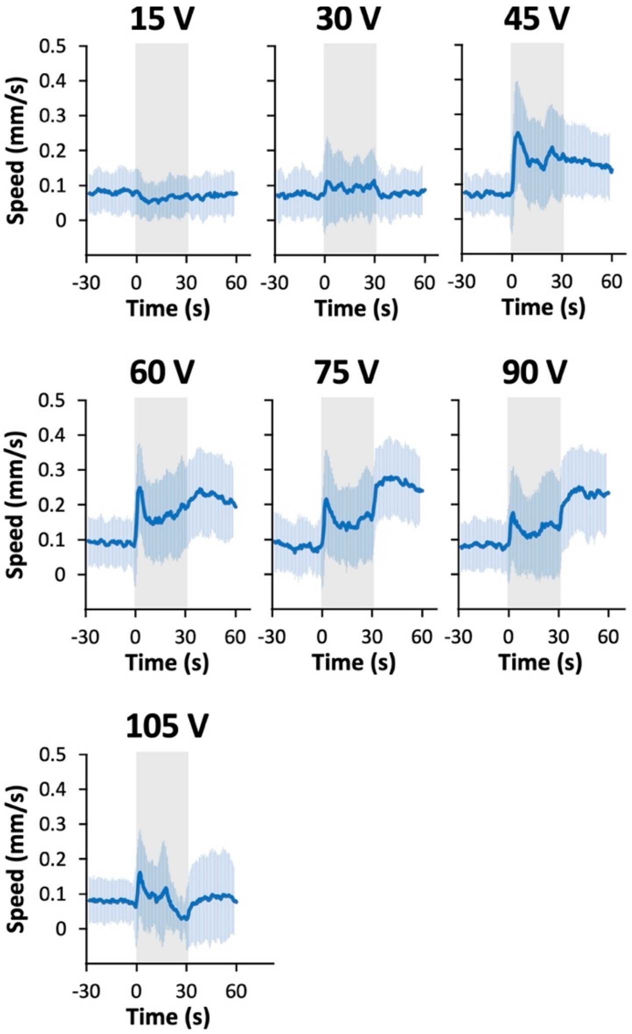

Figure S1. Speed-time graphs with different voltage stimulation at 60 Hz. Gray indicates the duration of electric stimulation (0-30 s). The thick line and the shaded region indicate the average ± SD. Sample numbers were 57–58 per condition, and the details are described in the Table S1.
