# Supplementary material for: Electric shock causes a fleeing-like persistent behavioral response in the nematode Caenorhabditis elegans
Source: Genetics. 2023 Aug 18;225(2):iyad148. doi: 10.1093/genetics/iyad148 (PMC10550322; doi:10.1093/genetics/iyad148)
Supplement: iyad148_Supplementary_Data [file iyad148_supplementary_data.zip › Figure_S2_GENETICS-2022-305494.pdf]

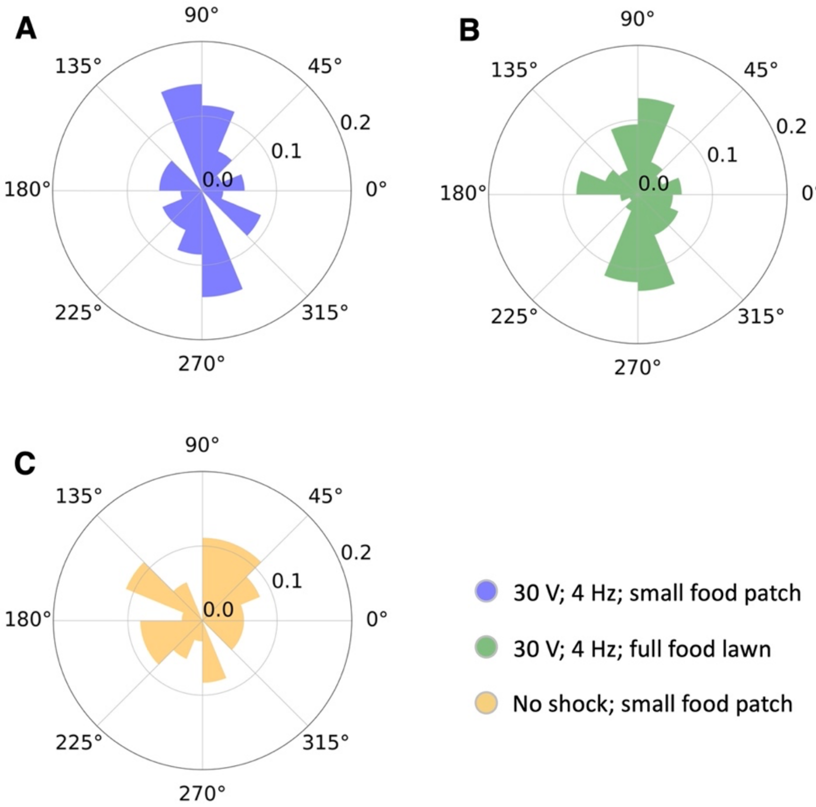

Figure S2. Movement directions of animals during the response. The angles of movement vectors from the beginning to the first 2 min of the stimulation were plotted. **A-C,** Rose plot for animals which were assayed on plate with small food patch (**A**, n = 35; Group 1) or full food lawn (**B**, n = 85; Group 2) with 30 V at 4 Hz, or small food patch without electric stimulation (**C**, n = 36; Group 3). Bin number for each chart is set at 16 bins. Statistical analysis performed is Watson U2 test, and *p* values for Groups 1 vs 2, 1 vs 3, and 2 vs 3 were all >0.1.
