# Supplementary material for: Electric shock causes a fleeing-like persistent behavioral response in the nematode Caenorhabditis elegans
Source: Genetics. 2023 Aug 18;225(2):iyad148. doi: 10.1093/genetics/iyad148 (PMC10550322; doi:10.1093/genetics/iyad148)
Supplement: iyad148_Supplementary_Data [file iyad148_supplementary_data.zip › Figure_S3_GENETICS-2022-305494.pdf]

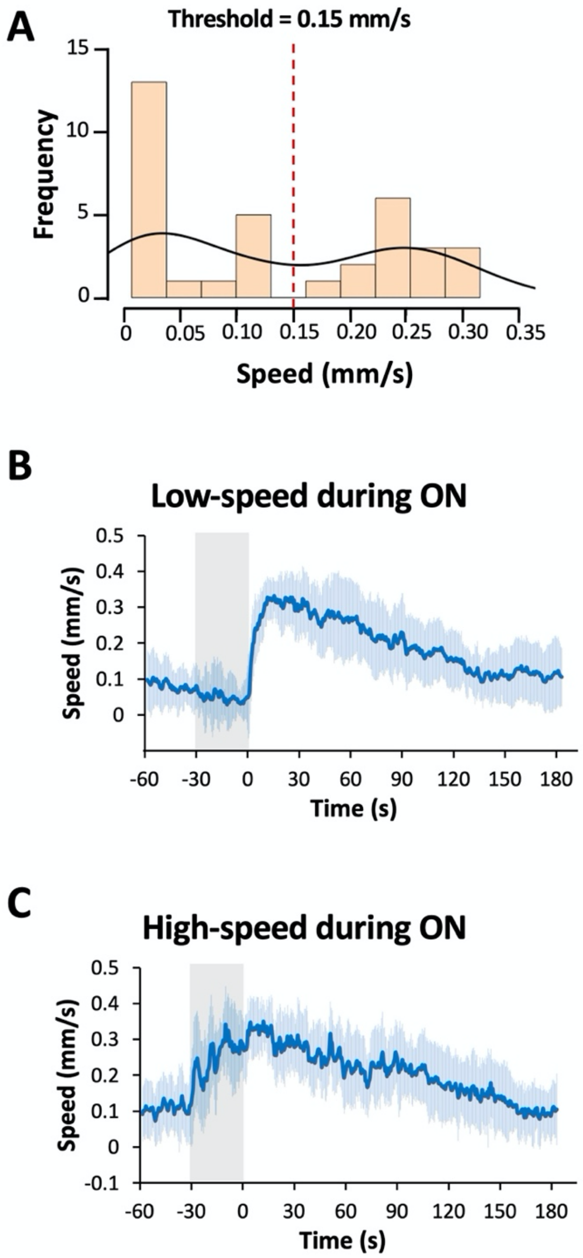

Figure S3. Low and high speed groups during 75 V stimulation. **A**, Histogram and its density (black line) indicates speed of each animal during the electric shock. From the histogram, we set the threshold as 0.15 mm/s to separate the low- (**B**) and high-speed (**C**) groups. Sample numbers were 20 and 15 for lower and higher speed groups, respectively, and the details are described in Table S1.
