# Supplementary material for: Electric shock causes a fleeing-like persistent behavioral response in the nematode Caenorhabditis elegans
Source: Genetics. 2023 Aug 18;225(2):iyad148. doi: 10.1093/genetics/iyad148 (PMC10550322; doi:10.1093/genetics/iyad148)
Supplement: iyad148_Supplementary_Data [file iyad148_supplementary_data.zip › Figure_S4_GENETICS-2022-305494.pdf]

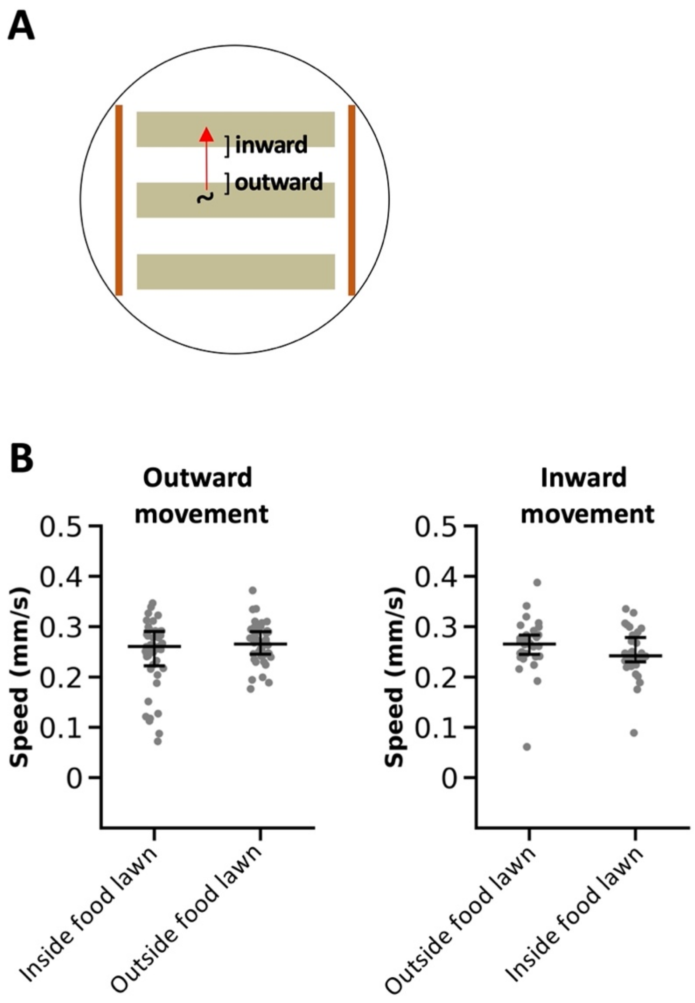

Figure S4. Worms' speed did not change when they move in or out of food. **A,** Illustration showing worms' movement across multiple food strips. When worms leave food strip and enter no food area, this movement is defined as "outward movement". When worms enter food strip from no food area, this movement is defined as "inward movement". **B,** Scatter plot showing average speed of individual animals with outward (left; n = 44) or inward (right; n = 32) movement during 30 V stimulation for 4 min. The average speed was calculated 10 s before and after the food exit/entry. Statistical analysis was performed by Wilcoxon signed-rank test, and no significant difference was observed.
