# Supplementary material for: Electric shock causes a fleeing-like persistent behavioral response in the nematode Caenorhabditis elegans
Source: Genetics. 2023 Aug 18;225(2):iyad148. doi: 10.1093/genetics/iyad148 (PMC10550322; doi:10.1093/genetics/iyad148)
Supplement: iyad148_Supplementary_Data [file iyad148_supplementary_data.zip › Figure_S5_GENETICS-2022-305494.pdf]

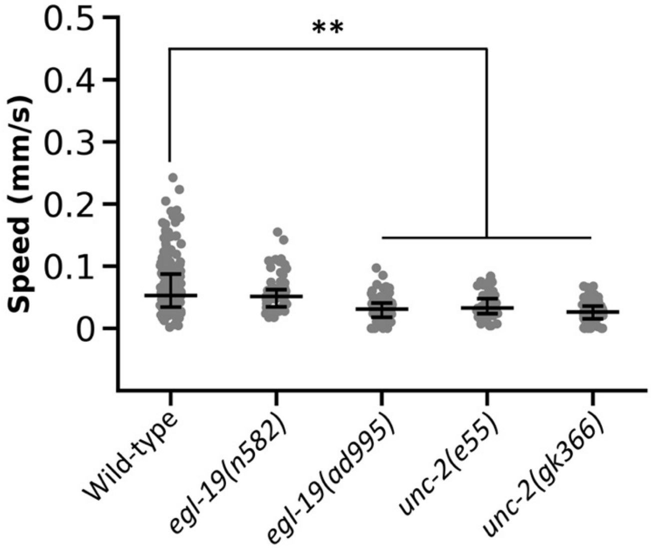

Figure S5. Basal speeds of wild-type and VGCC mutants before the 30 V and 75 V stimulations. Statistical values were calculated using Kruskal-Wallis test with Bonferroni correction. ** $p < 0.001$.
