# Supplementary material for: Electric shock causes a fleeing-like persistent behavioral response in the nematode Caenorhabditis elegans
Source: Genetics. 2023 Aug 18;225(2):iyad148. doi: 10.1093/genetics/iyad148 (PMC10550322; doi:10.1093/genetics/iyad148)
Supplement: iyad148_Supplementary_Data [file iyad148_supplementary_data.zip › Supplemental_Video_Legends_GENETICS-2022-305494.docx]

**Electric shock causes a fleeing-like persistent behavioral response in the nematode *Caenorhabditis elegans***

Ling Fei Tee, Jared J. Young, Ryoga Suzuki, Keisuke Maruyama, Sota Kimura, Yuto Endo, Koutarou D. Kimura

**Video S1.** Representative responses of animals in an assay plate subjected to 30 V stimulation at 4 Hz for 30 seconds. The trajectories and corresponding individual speed values produced by the animals were shown. The custom-made copper plate bridges were placed at left and right side of field view. The scale bars in all the videos represent 0.5 mm.

**Video S2.** Representative responses of animals in an assay plate subjected to 30 V stimulation at 4 Hz for 30 seconds. This video is from raw images used for the Video S1.

**Video S3.** Representative responses of animals in an assay plate subjected to 75 V stimulation at 4 Hz for 30 seconds. The trajectories and corresponding individual speed values produced by the animals were shown. One animal, but not the other three, increased speed during the electric shock while all the animals increased speed after the shock. Similarly to the Video S1, the custom-made copper plate bridges were placed at left and right side of field view.

**Video S4.** Representative responses of animals in an assay plate subjected to 75 V stimulation at 4 Hz for 30 seconds. This video is from raw images used for the Video S3.

**Video S5.** Representative responses of animals in a three stripe-food assay plate subjected to 30 V stimulation at 4 Hz for 30 seconds. The trajectories and corresponding individual speed values produced by the animals were shown. The custom-made copper plate bridges were placed at left and right side of field view.
