# Supplementary figures and images for: Electric shock causes a fleeing-like persistent behavioral response in the nematode Caenorhabditis elegans
Source: Genetics. 2023 Aug 18;225(2):iyad148. doi: 10.1093/genetics/iyad148 (PMC10550322; doi:10.1093/genetics/iyad148)

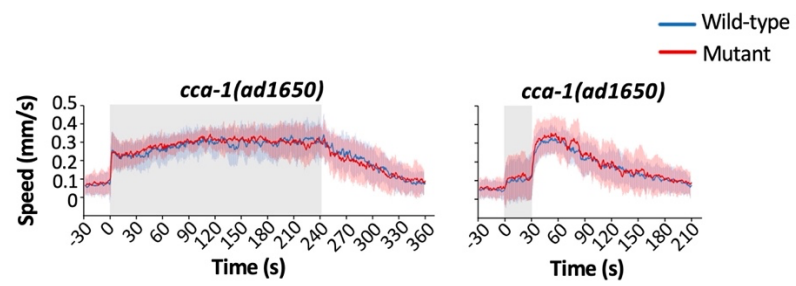

**Figure S6.** Speed-time graphs of ON (30 V, left) and OFF (75 V, right) responses of *cca-1*.

Supplement: iyad148_Supplementary_Data [file iyad148_supplementary_data.zip › Figure_S6_GENETICS-2022-305494.pdf]
